# Raltegravir use and outcomes among children and adolescents living with HIV in the IeDEA global consortium

**DOI:** 10.1002/jia2.25580

**Published:** 2020-07-28

**Authors:** Gabriela Patten, Thanyawee Puthanakit, Catherine C McGowan, Kara Wools‐Kaloustian, Rohan Hazra, Jorge A Pinto, Daisy Machado, Regina Succi, Annette H Sohn, Helena Rabie, Beverly Musick, Mary‐Ann Davies, IeDEA

**Affiliations:** ^1^ School of Public Health and Family Medicine Faculty of Health Sciences University of Cape Town Cape Town South Africa; ^2^ Department of Pediatrics Faculty of Medicine Chulalongkorn University Bangkok Thailand; ^3^ Research Unit in Pediatric Infectious Diseases and Vaccines Faculty of Medicine Chulalongkorn University Bangkok Thailand; ^4^ School of Medicine Vanderbilt University Nashville TN USA; ^5^ School of Medicine Indiana University Indianapolis IN USA; ^6^ Eunice Kennedy Shriver National Institute of Child Health and Human Development National Institutes of Health Bethesda MD USA; ^7^ School of Medicine Federal University of Minas Gerais Belo Horizonte Brazil; ^8^ Department of Pediatrics Escola Paulista de Medicina Universidade Federal de São Paulo/UNIFESP São Paulo Brazil; ^9^ TREAT Asia amfAR – The Foundation for AIDS Research Bangkok Thailand; ^10^ Department of Paediatrics and Child Health University of Stellenbosch Stellenbosch South Africa

**Keywords:** HIV, third‐line, raltegravir, integrase inhibitors, antiretroviral therapy, adolescents, children

## Abstract

**Introduction:**

As integrase inhibitors become available in low‐ and middle‐income countries (LMICs), they offer the potential to expand extremely limited treatment options available to children and adolescents. In LMICs, only small numbers have used raltegravir, primarily as part of third‐line regimens. Using data from the IeDEA global consortium, we aimed to describe the characteristics of children on raltegravir‐containing regimens and their outcomes.

**Methods:**

We included data from 1994 to 2017 from children (age <18 years), from East and Southern Africa, Asia and South America, who received cART regimens containing raltegravir for ≥90 days. We describe their characteristics at raltegravir start, and their immunological and virological outcomes.

**Results and discussion:**

In total, 62 children were included, with median age at raltegravir initiation of 14.3 years (IQR 11.2 to 15.8) and median CD4 count of 276 cells/µL (IQR 68 to 494). Among 40 (65%) with drug resistance testing prior to raltegravir, 71% were resistant to at least one protease inhibitor (PI), and 32% had high‐level resistance to at least one drug class. Most (n = 50; 81%) received raltegravir as part of third‐line cART following PI‐based regimens, and were on regimens containing four or more drugs (n = 47, 76%).

By database closure, median duration on raltegravir was 2.0 years (IQR 0.8 to 3.0), 1 (1.6%) patient had died, 6 (9.7%) were lost to follow‐up and 21 (34%) had discontinued raltegravir. Among 15 patients reporting reasons for stopping raltegravir, six discontinued because it was no longer available. Within one year of starting raltegravir, among 53 patients with VL measures, 40 (75%) had VL < 1000 copies/mL, and among 54 with a reported CD4 count, 45 (83%) and 36 (67%) were ≥350 and ≥500 cells/µL, respectively, with median CD4 count increasing to 517.5 cells/µL (IQR 288 to 810).

**Conclusions:**

Among children in LMICs, the initial use of raltegravir has been primarily for post PI‐based cART. We found good virological and immunological outcomes despite frequent prior triple‐class failure and high levels of drug resistance. Both access to raltegravir and long‐term adherence to regimens with large pill‐burdens remain challenging. Policies which promote earlier access to new drugs and simplify daily regimens for children and adolescents in LMICs are needed.

## 1 INTRODUCTION

Treatment options for children failing combination antiretroviral therapy (cART) in low‐ and middle‐income countries (LMIC) are extremely limited. With more children initiating cART as infants when protease inhibitor (PI)‐based regimens are recommended, and with an increasing number of older children surviving longer on cART, there is a growing need for third‐line and salvage options following PI‐failure.

New generation PIs, such as ritonavir‐boosted darunavir (DRV/r), as well as the integrase inhibitors (INSTIs) dolutegravir and raltegravir are slowly becoming available to children in LMICs, although their high costs greatly inhibits access. While dolutegravir offers the promise of a lower burden of treatment failure if used in first‐line and is a robust second‐ or third‐line option, it is currently not registered widely for children <6 years old or those <20 kg, whereas raltegravir is approved for use from birth. The 2019 World Health Organization (WHO) policy brief for first‐ and second‐line cART recommends raltegravir‐based regimens as an alternative first‐line for infants and children for whom approved dolutegravir dosing is not available, and as an alternative second‐line option, following failure of PI‐based cART [1].

Whilst raltegravir has been studied as a second‐line option in LMICs in adults [2], pharmacokinetic, safety and outcomes data on pregnant women, infants and children have largely been gathered through studies in the United States [3, 4, 5, 6]. Small numbers of children and adolescents in LMICs have been using raltegravir, primarily as part of third‐line regimens. The main objective of this multiregional analysis is to describe clinical, immunological and virological outcomes of raltegravir use among children and adolescents in the International epidemiology Databases to Evaluate AIDS (IeDEA) global consortium.

## 2 METHODS

### 2.1 Study population

Children and adolescents living with HIV with a record of being on raltegravir <18 years were eligible for inclusion. We excluded those with <90 days on raltegravir. Data from the previously described IeDEA multi‐regional collaboration of HIV cohort studies [7] were obtained from regions with eligible patients, namely IeDEA Southern Africa, East Africa IeDEA, IeDEA Asia‐Pacific and the Caribbean, Central and South America Network for HIV Research (CCASAnet). Each IeDEA site has institutional ethics approval to contribute data for IeDEA analyses. Informed consent was obtained when requested by local institutional review boards. The data centre at the University of Cape Town has ethics approval from the university’s Human Research Ethics Committee to combine and analyse these data. Routinely collected data were used from the date of cART initiation to database closure, which ranged between January and August 2017 across sites.

### 2.2 Study variables and analysis

We described the demographic, clinical, immunological and virological characteristics of patients at raltegravir initiation as well as their prior treatment history. We defined third‐line/salvage regimens as any regimen following exposure to PI‐based cART. We summarized the resistance profile of patients with genotypic resistance testing prior to raltegravir initiation, using the Stanford University HIV genotypic resistance interpretation [8, 9]. We described the resistance level to each drug and the most commonly reported clinically significant drug resistance mutations.

We described the immunological and virological outcomes for all patients and for those on raltegravir‐based third‐line regimens. Outcomes are reported at six months and one year from raltegravir initiation using the last available CD4 count or viral load (VL) measure in that time period. All analyses were performed in Stata version 15.0 (Stata Corporation, College Station, TX, USA).

## 3 RESULTS AND DISCUSSION

### 3.1 Patient characteristics

We identified 80 children on raltegravir, 11 were excluded because they received raltegravir for <90 days, and seven because they were on a clinical trial. Characteristics at cART and raltegravir initiation of the 62 patients included in our study are summarized in Table 1. The majority were adolescents, with a median age at raltegravir initiation of 14.3 years (IQR 11.2 to 15.8), with 3 (5%) being <6 years of age. Patients were fairly evenly distributed among the regions apart from East Africa which had only five patients. Median prior duration on cART was 8.6 years (IQR 6.1 to 10.8). The majority (n = 50; 81%) received raltegravir as part of a third‐line regimen following PI‐based cART. Almost a quarter (n = 15; 24%) had exposure to unboosted PI‐based regimens containing ritonavir (RTV) as the sole PI.

**Table 1:** Characteristics and outcomes of children and adolescents receiving raltegravir as part of combination ART (n = 62)

	n (%)
Characteristic at cART initiation	
Region	
CCASAnet	18 (29.0%)
Asia	21 (33.9%)
Southern Africa	18 (29.0%)
East Africa	5 (8.1%)
Female	37 (59.7%)
Age in years (median, IQR)	3.9 (0.8 to 8.4)
Year of cART start	
1994 to 1999	7 (11.3%)
2000 to 2004	22 (35.5%)
2005 to 2009	26 (41.9%)
2010 to 2015	7 (11.3%)
Characteristics at raltegravir initiation	
Age in years (median, IQR)	14.3 (11.2 to 15.8)
Year of raltegravir start	
2006 to 2009	7 (11.3%)
2010 to 2013	22 (35.5%)
2014 to 2016	33 (53.2%)
Years on cART (median, IQR)	8.6 (6.1 to 10.8)
CD4% (median, IQR, n)	15 (5.1 to 22.2), 44
CD4 (median, IQR, n)	276 (68 to 494), 50
log_10_ HIV viral load (median, IQR, n)	4.7 (3.7 to 5.2), 46
cART history prior to raltegravir	
PI‐based cART only	19 (30.6%)
NNRTI‐ followed by PI‐based cART	25 (40.3%)
PI‐followed by NNRTI‐based cART	6 (9.7%)
NNRTI‐based cART only	5 (8.1%)
Non‐standard cART regimens	2 (3.2%)
Unknown	5 (8.1%)

IQR, inter‐quartile range; NNRTI: non‐nucleoside reverse transcriptase inhibitors; PI, protease inhibitors.

Among 50 patients with recorded CD4 count at raltegravir start, the median was 276 cells/µL (IQR 68 to 494), 23 (46%) were <200 and 16 (32%) were <100.

### 3.2 Resistance profile

Prior to initiating raltegravir‐based regimens, 40 patients (n = 62, 65%) had undergone resistance testing, of whom 38 had data on PI resistance, 39 on nucleoside reverse transcriptase inhibitor (NRTI) and NNRTI resistance, and two on INSTI resistance.

**Figure 1:**
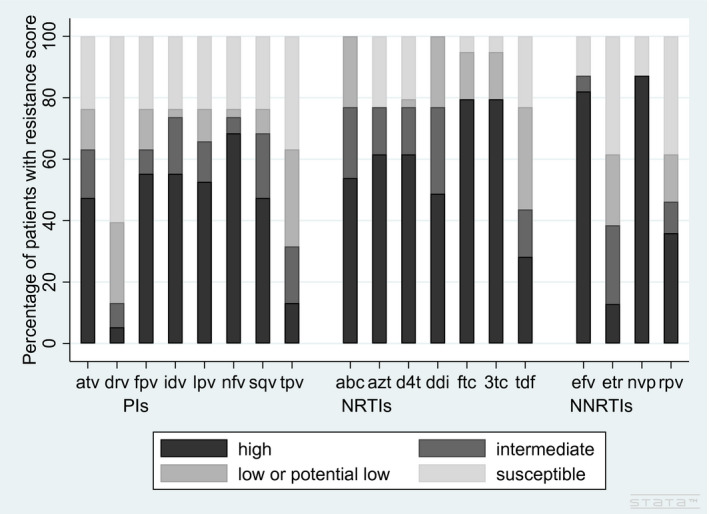
Proportion of children and adolescents living with HIV with resistance testing prior to raltegravir, with resistance to specific antiretroviral drugs. Resistance scores according to the Stanford University HIV genotypic resistance interpretation algorithm [8], grouped according to drug class. Protease inihibitors: atv (atazanavir/ritonavir), drv (darunavir/ritonavir), fpv (fosamprenavir), idv (indinavir), lpv (lopinavir/ritonavir), nfv (nelfinavir), sqv (saquinavir), tpv (tipranavir). Non‐nucleos(t)ide reverse transcriptase inhibitors: abc (abacavir), azt (zidovudine), d4t (stavudine), ddi (didanosine), ftc (emtricitabine), 3tc (lamivudine), tdf (tenofovir). Nucleoside reverse transcriptase inhibitors: efv (efavirenz), etr (etravirine), nvp (nevirapine), rpv (rilpivirine).

Both patients with INSTI resistance data were susceptible to all drugs in the class. Figure 1 describes the proportion of patients with resistance to each drug for the remaining drug classes. Twenty‐seven (71%) patients had high‐level resistance to at least one PI, 38 (97%) had high‐level resistance to at least one NRTI and 34 (87%) had high‐level resistance to at least one NNRTI (all being highly resistant to nevirapine). Twelve patients (30%) were highly resistant to all drugs in at least one drug class: seven to all NRTIs, two to all PIs and five to all NNRTIs tested. Of these patients, 11 had VL measures within six months of starting raltegravir and 10 (91%) were <1000 copies/mL.

Among the 38 with PI resistance data, one patient was susceptible to all PIs, 20 (53%) had high‐level resistance to lopinavir/ritonavir (LPV/r) and 23 (61%) had high or intermediate resistance to both atazanavir and LPV/r. Of the 27 (71%) with high‐level resistance to at least one PI, seven were also highly resistant to all NRTIs and four highly resistant to all NNRTIs. Ten (26%) patients had low‐level, three (8%) intermediate and two (5%) high‐level resistance to DRV/r. The two patients with high‐level resistance to DRV/r had high‐level resistance to all other PIs and were resistant to all NNRTIs.

**Figure 2:**
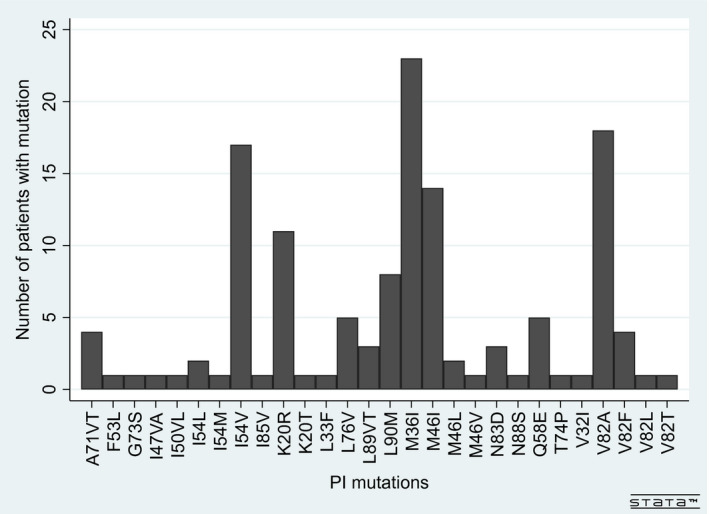
Protease inhibitor mutations detected among children and adolescents living with HIV with resistance testing prior to raltegravir (n = 38).

The most commonly reported PI drug resistant mutations are shown in Figure 2. Twenty‐two (56%) patients had type I TAMs and 28 (74%) had type II TAMs. The most common NNRTI mutations were K103NS (19, 49%) and K101EHPQ (13, 33%).

### 3.3 Treatment outcomes on raltegravir

At database closure, the median duration on raltegravir was 2.0 years (IQR 0.8 to 3.0), 1 (1.6%) patient had died, 6 (9.7%) were lost to follow‐up, and 21 (34%) had discontinued raltegravir. Among the 15 patients with recorded reasons for raltegravir discontinuation, six stopped because it was no longer available (2 from each of the African and Asia‐Pacific regions), four because of treatment failure (virological, immunological or due to resistance), three due to side effects or drug‐specific toxicity, one related to patient decision and one to switch to more effective treatment. Among the six who stopped because of lack of availability, two were placed on dolutegravir and the remaining four returned to PI‐based regimens.

Treatment outcomes for all patients and those on raltegravir as part of third‐line regimens are summarized in Table 2. Raltegravir was most commonly combined with PIs and NRTIs (n = 42, 68%), the most common regimen being raltegravir with DRV/r and NRTIs (n = 36, 50%). Most patients were on regimens containing four drugs (n = 28, 45%), with 19 (31%) patients on regimens containing five or more drugs (including raltegravir and counting RTV separately when used as a separate drug to boost a PI). At database closure three patients had switched to dolutegravir following raltegravir, all from Southern Africa.

**Table 2:** Drug regimens, immunological and virological outcomes for children and adolescents living with HIV on raltegravir‐based ART, overall and for those on raltegravir as part of a third‐line regimen (n = 62)

	All	Third‐line
	n = 62	n = 50
Raltegravir‐based ART regimen		
Raltegravir + NRTIs	5 (8.1%)	4 (8.2%)
Raltegravir + PI + NRTIs	42 (67.7%)	33 (66.0%)
Raltegravir + NNRTI + NRTIs	1 (1.6%)	1 (2.0%)
Raltegravir + PI + NNRTI (+ NRTIs)	10 (16.1%)	8 (16.3%)
Raltegravir + other drug classes	4 (6.5%)	4 (8.2%)
Duration on raltegravir (years)	2.0 (IQR 0.8 to 3.0)	1.9 (IQR 0.8 to 2.6)
CD4 at raltegravir start (median, IQR, n)	275.5 (65 to 494), 50	210 (62 to 531), 43
Immunological and virological outcomes		
Within six months		
CD4 (median, IQR, n)	396 (211 to 634), 50	382 (211 to 666), 43
CD4 ≥ 350	43 (86%)	34 (79.1%)
CD4 ≥ 500	32 (64%)	24 (55.8%)
Viral load <400	35 (73%), 48	31 (73.8%), 42
Viral load <1000	37 (77%), 48	33 (78.6%), 42
Within 12 months		
CD4 (median, IQR, n)	517.5 (288 to 810), 54	496 (288 to 869), 46
CD4 ≥ 350	45 (83%)	35 (76.1%)
CD4 ≥ 500	36 (67%)	27 (58.7%)
Viral load <400	37 (70%), 53	32 (71.1%), 45
Viral load <1000	40 (75%), 53	35 (77.8%), 45

Other drug classes include CCR5 receptor antoagonists and HIV fusion inhibitors. CD4 count in cells/µL, viral load (VL) in copies/mL. IQR, Inter‐quartile range; NNRTI, non‐nucleoside reverse transcriptase inhibitors; NRTI, nucleoside reverse transcriptase inhibitor; PI, protease inhibitors.

### 3.4 Virological outcomes

Among 53 patients with VL measures within 1 year of starting raltegravir, 40 (75%) were <1000 copies/mL and 37 (70%) were <400. Among the 11 who failed to suppress and with VL > 1000 on initiating raltegravir, seven (64%) experienced reductions in VL, with median reduction 5.2 log (IQR 3.9 to 5.4), and four (36%) had increasing VL. Similar rates of virological suppression were experienced by those initiating raltegravir as part of third‐line regimens (Table 2). At database closure, among 46 who were virologically suppressed at any time whilst on raltegravir, eight (17%) experienced virological rebound to VL > 1000, with a median time to rebound from starting raltegravir of 391 days (IQR 201–519).

### 3.5 Immunological outcomes

Among 54 patients with a CD4 count within one year of starting raltegravir, 45 (83%) and 36 (67%) were ≥350 and ≥500 cells/µL, respectively, with median CD4 count increasing by 242 to 517.5 (IQR 288 to 810). Within 1 year on raltegravir, among 36 patients who started with CD4 < 500, median CD4 count increased from 70 (IQR 22 to 210) to 285 (IQR 106 to 382); 15 (42%) recovered to CD4 ≥ 500 and 23 (64%) had CD4 ≥ 350. However, three (8%) experienced declining CD4, with declines of between 3 and 264. All 11 patients with CD4 ≥ 500 at raltegravir start maintained CD4 ≥ 500 within one year. For those on third‐line, although the median CD4 count at raltegravir start and within one year was lower, there was a similar increase of 286 in median CD4 count to 496 (IQR 288 to 869) (Table 2).

This study represents the largest reported cohort of children receiving raltegravir as part of routine care in LMICs. Raltegravir was primarily used following failure of PI‐based regimens, initiated during adolescence, after long durations on cART, with almost half of children being immunocompromised. Despite high levels of drug resistance to other classes prior to raltegravir, there were good virological outcomes with three‐quarters of patients suppressing within one year. Median CD4 count substantially increased and most patients experienced immune recovery or maintained CD4 ≥ 500 cells/µL. Notably, three‐quarters of patients received raltegravir as part of regimens containing four or more drugs. This large pill‐burden and the fact that raltegravir is taken twice daily, possibly contributed to difficulties with long‐term adherence, with close to 20% of patients experiencing viral rebound following suppression. This is concerning and raises the questions of whether poor adherence to raltegravir could compromise future use of dolutegravir. Moreover, many of those stopping raltegravir did so due to lack of availability, demonstrating local challenges to sustaining access to less commonly used antiretrovirals.

The most recent WHO guidelines recommend INSTI‐based second‐line for children and infants following failure of first‐line PI‐based cART. Our study provides some of the initial evidence of the use of raltegravir for this purpose, with a third of patients having had prior exposure to only PI‐based cART. Our results show that raltegravir provided effective treatment for children with few alternatives. Half our patients had prior treatment with both NNRTI‐ and PI‐based cART. Almost a quarter had exposure to RTV‐only based cART, now known to select for major PI resistance mutations [10]. High levels of drug resistance were found, 60% having high or intermediate resistance to the most commonly recommended PIs for use in children, and almost 40% having some recorded resistance to DRV/r.

While there are studies on the use of INSTIs and third‐line regimens among children and adolescents in LMICs, they have included only small numbers of patients. A study of 12 heavily pre‐treated French adolescents who were treated with raltegravir, DRV/r or etravirine and a Spanish study of 23 multidrug‐resistant paediatric patients who received etravirine both reported good virological outcomes with few side effects [11, 12]. Two studies from Southern Africa, one of four children from Botswana and another of 35 treatment‐experienced children from South Africa, including 25 who received raltegravir, both found good virological outcomes [13, 15]. A Thai study of 54 adolescents on third‐line regimens, including 2 on raltegravir, found that 66% achieved VL < 50 copies/mL, but close to one third had VL > 1000 attributed to poor adherence [14].

Our study is limited by the use of routinely collected data for a small number of children from different regions where eligibility criteria for raltegravir, and prior drug resistance testing varied. We did not have data on drug formulations, adherence to raltegravir or on measures taken to address adherence barriers across the different regions.

## 4 CONCLUSIONS

Among adolescents and children in LMICs, raltegravir provided a much‐needed treatment option for children failing PI‐based first‐ and second‐line cART. We found good virological and immunological outcomes despite frequent prior triple‐class failure and high levels of drug resistance, but accessing this drug remains challenging and pill burden was high. Policies which promote earlier access to new drugs for children and adolescents in LMICs to improve treatment outcomes and simplify daily regimens are needed.

## COMPETING INTEREST

The authors declare that they have no competing interests.

## AUTHORS’ CONTRIBUTIONS

GP undertook the data compilation and analysis, generated the tables and figures, interpreted the results, wrote all drafts and finalized the manuscript. TP, CM, KW‐K, RH, JP, DM, RS, AS, HR, BM and M‐AD managed the cohort and transferred routinely collected data. All authors were involved in designing the study and reviewed the final manuscript.
